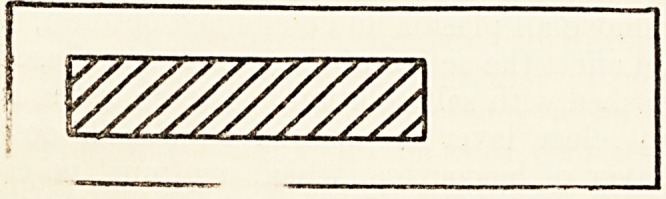# The Actual Technique of Tuberculo-Opsonic Estimation

**Published:** 1908-12-12

**Authors:** 


### Pathology.

### THE ACTUAL TECHNIQUE OF TUBERCULO-OPSONIC ESTIMATION.

In dealing with this subject no excuse is required for going into elementary details of procedure, because it is on such that the reliability of all technique depends. It may be added that in the following account small immaterial modifications are made in order to suit a worker who has to depend upon individual resources. Most of the apparatus required can be made from glass tubing, either quarter-inch bore, or what is known as "quill" tubing, both supplied in three-feet lengths by dealers in laboratory accessories. By means of a brazier's lamp and a three-cornered file, the former is fashioned into pipettes (fitted with a rubber teat like a fountain-pen filler), and the latter into blood capsules, one end of which is to be drawn out rather finer and longer than the other, thus:—

Actual Size.

Two of these capsules are taken and filled in the following way, the one with blood of the patient, the other with that of a normal individual. The skin at the base of the thumb-nail is wiped over with spirit, which is then allowed to evaporate, and a band having been wound tightly round the first phalanx, a smart jab is given with a sterilised needle into the prepared area. Sir Almroth Wright's direction is: "Prick yourselves generously." The capsule being held below the bleeding point, with the shorter, wider capillary end applied to it, blood flows down into the bulb of the capsule. When this is about a third full the end must be withdrawn, and the longer limb passed once or twice over a spirit-lamp flame, which is then used to seal it up. The retreat of the blood into the bulb of the capsule is now much accelerated, and the shorter end can be likewise closed. Throughout care must be taken not to char the blood. The capsules are now to be stood up and left aside while the blood in them clots.

To obtain *washed corpuscles* use tubes made by sealing up in the brazier's lamp flame 5-in. sections of the larger bore tubing. Make with a file two marks, one 3½ in., the other 4 in. up the tube. By means of a pipette (washed out first with water), fill up to the first with hypertonic solution—1 per cent. sodium citrate in .85 per cent. saline solution, conveniently made with Parke, Davis and Co.'s tabloids C.T. No. 476. Then, pricking oneself in the way before described, add direct from the thumb blood up to the second mark. Mix well, and at once, inverting the tube over the bleeding point on the back of the thumb. Then centrifuge at top speed for 15 minutes, counterweighting with a similar tube filled with water.

*Emulsion.*—Clean a small agate mortar with spirit and let it dry by evaporation. Put one platinum loopful of dried tuberculin into the mortar, and rub it down, dry as it is, until quite smooth. Then add with a pipette a drop of .1 per cent. saline solution (got by diluting .85 per cent. saline solution) and rub well again. Repeat until 15 drops have been added. To get a satisfactory emulsion this procedure should occupy 20 minutes. Finally, the emulsion is put into a small glass tube and centrifuged at gentle speed for two minutes.

We return now to the *washed corpuscles.* After the tube containing them is taken from the centrifuge its appearance is diagrammatically thus:—

1 and 2......First and second marks.

3......Solution and plasma.

4......Pellicle of leucocytes.

5......Red Blood cells.

To remove all plasma and contained opsonin, which would affect the action of the leucocytes, these must be washed with salt solution. Therefore the supernatant clear layer is pipetted off almost down to the layer of leucocytes, which one must be careful not to disturb. The place of this supernatant layer is supplied by .85 saline solution, and the tube is shaken up and centrifuged as before for 15 minutes, although it is well to stop when the circumference of the pellicle of leucocytes begins to separate from the side of the tube. On removal from the centrifuge its appearance is much as in the last diagram. In place, however, of again removing the supernatant fluid, one has now to lower carefully through it the point of a pipette down on to the leucocytes, and to draw them up by gentle manipulation of the rubber teat. This is a ticklish operation, and the tube should be held steadily on some firm substance in a good light. To start with, the teat should be held at the top and completely collapsed by steady pressure. Some red cells must necessarily come too. Express into a clean watch glass, mix by drawing up and down once or twice, and then cover over. Lastly, the capsules, in which the blood before this has clotted, must be centrifuged at top speed for two or three minutes. The serum becomes supernatant, and the capsules may be opened by nicking off the top with a glazier's diamond.

There are now the three essentials: (1) emulsion, (2) washed corpuscles, (3) serum in the capsules. To mix equal volumes of these, mark off one inch, say, of the thin extremity of a pipette with a grease-pencil, and flame so as to fix the mark. Dip well into the emulsion (so as to avoid taking the top layer) and draw up to the mark. Then remove and admit a bubble of air, which serves to separate the emulsion from the equal volume of serum which follows. Draw serum up to the mark, and similarly separate from a volume of washed corpuscles. All this is much easier if the end of the pipette covered by the rubber teat is almost sealed up, so as to admit air slowly. Express on to a clean light-coloured glazed surface, and draw up and down once or twice to mix well. Finally, draw up the mixture into the pipette, seal its end in the flame, taking care not to heat the contents, and put into the incubator at 37.5° C. for 15 minutes.

*Mounting and Staining.*—The slides, which for convenient identification may be numbered with a glazier's diamond, must be absolutely clean and grease-free. Slides when finished with should have the cedar-wood oil wiped off with spirit, and should be kept in spirit. To clean, rub with cigar-ash on a muslin cloth slightly moistened with that fluid. Then wipe off the ash with a grease-free cloth (old handkerchiefs boiled in washing soda and water). When the pipette has been the exact time in the incubator remove it, open it, and express the contents, mixing well as before. Then put a tiny drop near the end of the slide, and make a film by means of another slide held at an angle of 30° with the first. The film should have the following position relatively to the slide:—

Always make two films of each blood. After drying in air fix by passing once quickly through the flame. Stain films all together in a tube containing hot carbol fuchsin, for ten minutes; wash in water, decolorise in 3 per cent. (by volume) sulphuric acid in methylated spirit, wash again, put in 4 per cent. acetic acid solution, and after washing into ammonia solution (three or four drops to a pint of water). Then wash and stain in Löffler's blue for 20 seconds.

*Counting.*—Look first with the low power at the edge of the distal part of the film. Having found leucocytes, clamp the slide down and examine with the oil immersion. Only polynuclears (not eosinophiles) are to be reckoned with. Count the number of bacilli in ten leucocytes, and then note down on paper, going on until 100 have been counted. If the number of bacilli, however, is low, it is well to count, say, 100 of the latter. There is a variety of rules in vogue respecting leucocytes containing a clump of bacilli, or a greater number than five or six. It is perhaps best to neglect the first class, and to count the second as only containing the limiting number taken. Whatever rules are adopted, they must be applied impartially to the control as well as to the other films. The opsonic index, of course, is represented by a fraction whose numerator is the number of bacilli ingested by leucocytes of the patient's blood, and whose denominator is the number ingested by leucocytes of the blood of the control. The usual limits lie between .6 to 1.7.

## Figures and Tables

**Figure f1:**
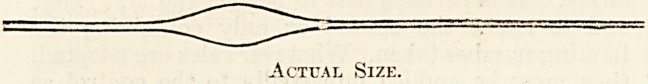


**Figure f2:**
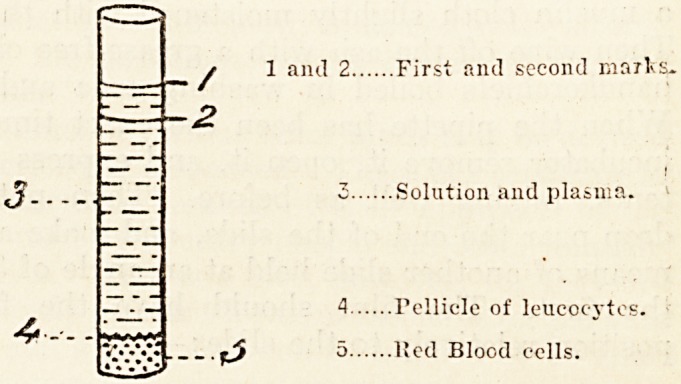


**Figure f3:**